# Forming Spacers *in Situ* by Photolithography to Mechanically Stabilize Electrofluidic-Based Switchable Optical Elements

**DOI:** 10.3390/ma9040250

**Published:** 2016-03-30

**Authors:** Meihong Wang, Yuanyuan Guo, Robert A. Hayes, Danqing Liu, Dirk J. Broer, Guofu Zhou

**Affiliations:** 1 Electronic Paper Display Institute, South China Normal University, Higher Education Mega Center, Guangzhou 510006, China; 2013022268@m.scnu.edu.cn (M.W.); guoyy@scnu.edu.cn (Y.G.); guofu.zhou@guohua-oet.com (G.Z.); 2 Eindhoven University of Technology, Institute for Complex Molecular Systems, Eindhoven 5612AP, The Netherlands; d.liu1@tue.nl (D.L.); d.broer@tue.nl (D.J.B.); 3 Shenzhen Guohua Optoelectronics Tech. Co. Ltd., Shenzhen 518110, China; 4 Academy of Shenzhen Guohua Optoelectronics, Shenzhen 518110, China

**Keywords:** electrofluidic display, electrowetting, spacer, phase separation, photopolymerization, mechanical stability

## Abstract

Electro-Fluidic Displays (EFD) have been demonstrated to be an attractive technology for incorporation into portable display devices. EFDs have excellent optical efficiency and fast switching enabling video content. Ensuring mechanical stability of EFD display cells is a key challenge and essential for developing large area as well as flexible displays. Although the electro-optic performance of an EFD, unlike a liquid crystal display (LCD), is insensitive to cell-gap, extreme changes in cell-gap can result in irreversible collapse of the cell. Here we use photolithography to develop spacers to prevent cell-gap collapse and provide the required mechanical stability for EFD devices. The spacer is formed directly on the cover plates (ITO/glass) after cell assembly with UV light induced phase separation polymerization in the illuminated area. Phase separation behavior between polar aqueous solution and polymer is closely related to the solubility of acrylate monomers. In this work, polyethylene glycol diacrylate (PEGDA) as cross-linker, 2-hydroxyethyl acrylate (HEA) and acrylic acid or acrylamide as co-monomers are investigated for fabricating the spacers. PEGDA was added to the mixtures in order to increase the mechanical strength of the spacer. The spacers showed excellent performance for cell-gap control in EFD devices.

## 1. Introduction

**Figure 1:**
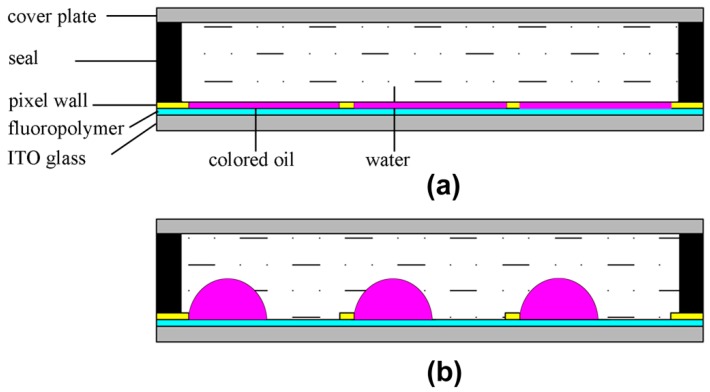
Electro-Fluidic Display (EFD) cell architecture in the (**a**) colored “off”; and (**b**) transparent “on” states.

The electrowetting effect is usually ascribed to Lippmann [1] who in 1875 made an early electrocapillary investigation. The modulation and reversibility of electrowetting on bare electrodes is usually limited by electrolysis when a few hundred millivolts are applied. In the 1990s, Berge [2] improved performance greatly by introducing an insulator layer to separate the conductive liquid from the metallic electrode, so-called electrowetting on dielectric (EWOD). In this way device electrowetting modulation and switching reliability could be significantly improved. Applying the voltage across the insulator/water interface changes the distribution of charges and decreases the contact angle of the droplet on the insulator layer [3,4]. In other words, the hydrophobicity of the insulator layer is reduced by the applied voltage. The electrowetting principle has spawned many interesting applications including lab on chip that allows for chemical reactions [5,6], splitting and mixing [7,8] and detection [9,10] of microdroplets as well as microelectromechanical systems (MEMS) like switches, latching relays, micropumps [11,12], adjustable microlenses [13] with a variable focal length and fiber optics [14,15] for switching light. Hayes and Feenstra [16] prototyped ePaper displays based on electrowetting. Low power consumption, sunlight readability, wide viewing angle and switching speed are the collective advantages of Electrowetting Displays (EWD)/EFD [3,16,17] over other reflective display technologies. The technology is suitable for a wide range of display applications from smart wearables to large-area outdoor display screens. The basic architecture consists of a pixelated substrate electrode, a hydrophobic insulator layer, pixel wall that confines the colored oil, the two fluid layers (non-polar colored oil and polar aqueous solution) and the common electrode. The device is sealed with an adhesive at the cell edge. Without electric field, the oil film wets the hydrophobic insulator layer, showing the oil’s color (Figure 1a). When a voltage is applied, the colored oil will be moved into the corner of the pixel, as the water wets the insulator. In this manner the pixel can become up to 85% transparent and ambient light reflects from the external white reflector (Figure 1b). 

**Figure 2:**
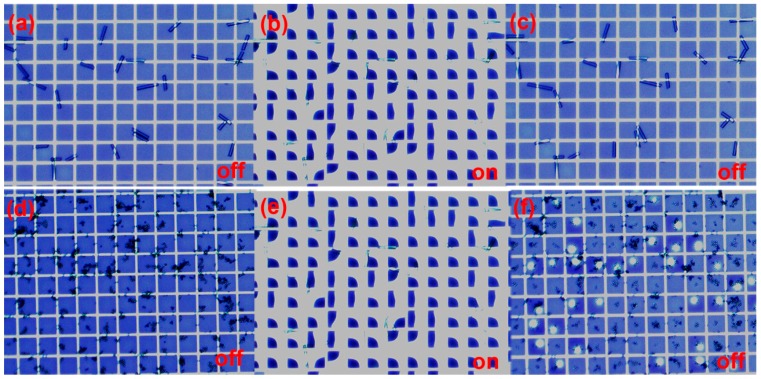
These images show an off/on/off switching cycle with and without voltage (30 V) after adding the rod (**a**–**c**)) and sphere (**d**–**f**) spacers into our EFD panels. The rod spacers (diameter: 30 μm, length: 90–220 μm) were supplied by Nippon Electric Glass Co., Ltd. (Osaka, Japan) The sphere spacers (average particle diameter: 15 μm ± 0.1 μm) were supplied by Sekisui Chemical Co., Ltd (Osaka Japan). The pixel size was 150 μm. The rod spacers interfered less with the fluidic motion as in most cases they were able to bridge the pixel wall structure and not contact the oil/insulator interface.

For large area or flexible displays, mechanical stability and maintaining a finite cell-gap between the two substrates are critical factors to prevent external destabilization by pressure, bending, and mechanical shock. Introducing spacers into the display is an obvious way to stabilize the cell gap. In liquid crystal displays (LCD), where extremely good control of cell gap (typically 2 μm) is required, spherical or rod spacers are typically employed. In an LCD there is no pixel wall and the liquid crystals are filled directly into the cell. Spacers can be dispersed into the cell randomly. In EFD devices the cell gap is much larger (typically 50–100 μm) and this methodology is not so suitable, in particular for spherical spacers which would occupy significant aperture (Figure 2). Besides, unlike LCD, we would prefer to position the spacers over pixel walls instead of the oil areas, so that the spacer will not disturb the colored oil motion. In current EFD devices cell gap collapse is prevented by either increasing substrate thickness, or associated rigidity, or by increasing the edge seal adhesive thickness. However, neither is desirable in the longer term where larger displays and flexible substrates will be required. In our research, we have developed a novel spacer solution for EFD using photo-induced phase separation polymerization. It is an *in situ* single step process to form spacers in the selected areas in a simple additive manner rather than conventional photolithography. Previous work has used this method to develop the polymer walls to isolate the liquid crystals in mixtures for enhancing mechanical stability [18,19,20,21,22,23]. Phase separation was first reported for constructing polymer dispersed liquid crystal (PDLC) films in 1986. It involved initiating the polymer precursor by heat or light, cooling, or solvent evaporation [24,25,26]. Among them, photo induced polymerization, which utilizes light energy to initiate chain reactions to synthesize polymer materials, offers a number of advantages: solvent-free formulations, low energy input, room temperature treatment and low cost. This technology offers a quick and effective transformation method from monomer into a cross-linked polymer with tailored mechanical properties. Different structures can also be created simply with patterned masks. Differences in the monomer reactivity, size and cross-linking ability, result in gradients in the monomer chemical potentials [27,28,29]. These chemical potentials provide the driving force for monomer migration and for polymer formation in the illuminated regions [18,22,30,31]. 

For EFD devices, the cell is filled with water and the cell gap is large, necessitating a novel approach. All of the monomers, cross-linkers, photoinitiator and photoinhibitor need to be water soluble UV curable materials. Although there are many UV water soluble materials, some drawbacks still limit their application given the requirements in EFD devices. Firstly the mixed solution should be colorless. Additionally, materials are required to dissolve in solution at room temperature and neither dissolve nor react with the colored oil in the pixel and the edge seal adhesive. In our research, spacers were polymerized in the selective area based on PEGDA incorporation with acrylate monomers. PEGDA consists of a linear PEG backbone with one acrylate group attached to each end of the PEG chain and its chemistry is highly tunable. PEGDA has been extensively investigated as a scaffold in tissue engineering due to its ability to withstand bending deformations [32,33,34,35]. The aim of this work is to develop a robust methodology to provide good mechanical stability for EFD devices.

## 2. Results and Discussion

### 2.1. Swelling of PEGDA Hydrogels

**Figure 3:**
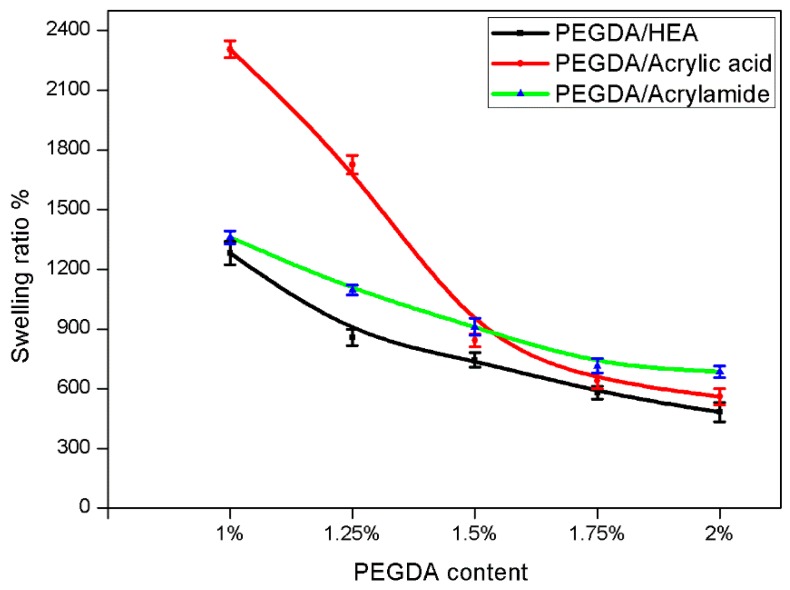
The swelling ratio of polyethylene glycol diacrylate (PEGDA) cross-linked hydrogels with different monomers. The error barsshow mean and standard deviation.

Hydrogels can swell to a considerable extent in water solutions. The swelling behavior of PEGDA hydrogels was investigated as a function of PEGDA content. The mesh size and swelling ratio of the hydrogels can be controlled by the different molecular weight of the polymer. Larger polymer molecular weight corresponds with larger mesh size [36,37]. The swelling ratio increases as the PEG molecular weight increases. In view of the mechanical requirements for spacers in our EFD devices we chose the lower molecular weight of PEGDA (*MW* = 250). Figure 3 quantitatively illustrates how the swelling ratio decreased with increasing PEGDA content. This is because the degree of cross-linking affects the swelling ratio of the hydrogels, the higher the degree of cross-linking with increasing the PEGDA content, the more difficult for water molecules to penetrate into the hydrogel network. Figure 3 shows that the swelling ratio of PEGDA/HEA hydrogels is less than PEGDA/acrylic acid and PEGDA/acrylamide hydrogels. This could be attributed to the more complete hydrolysis and stronger water uptake ability of acrylic acid and acrylamide. 

### 2.2. Mechanical Properties

Mechanical properties are strongly influenced by the swelling ratio and the degree of cross-linking. The improvement in the mechanical properties can be attributed to the formation of a dense network that can sustain high mechanical deformation. At a lower swelling ratio, the polymer chains are close to each other. The interaction between polymer chains in hydrogels is reinforced, and the hydrogel exhibited good mechanical strength. In contrast, at a higher swelling ratio, the hydrogel network is diluted, and the interaction is weakened. Thus, the strength of hydrogels decreased.

**Figure 4:**
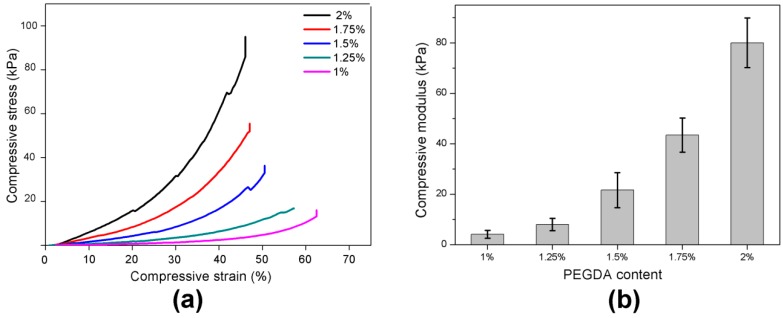
Mechanical properties of PEGDA (polyethylene glycol diacrylate)/HEA (2-hydroxyethyl acrylate) hydrogels were evaluated using an unconfined compression test. (**a**) Stress strain curves for different PEGDA content are shown; (**b**) An increase in compressive modulus with PEGDA content indicated the formation of a cross-linked network. The error bars show the standard deviation from the mean.

Mechanical properties of PEGDA hydrogels were determined by unconfined compression tests with the mechanical tester. Figure 4a demonstrates stress-strain curves of hydrogels swollen to maximum water content (no inhibitor) with different PEGDA content. The material is biphasic and as such the modulus increases with compression as shown in [38]. The stiffness of the hydrogel networks was determined by evaluating the compressive modulus from the gradient of the stress-strain curves in the linear “toe” region (5%–15%) [38]. The compressive modulus increased from 4 ± 2 kPa to 80 ± 10 kPa when PEGDA content increases from 1% to 2% (Figure 4b).

### 2.3. Influence of Exposure Time on the Polymerization

**Figure 5:**
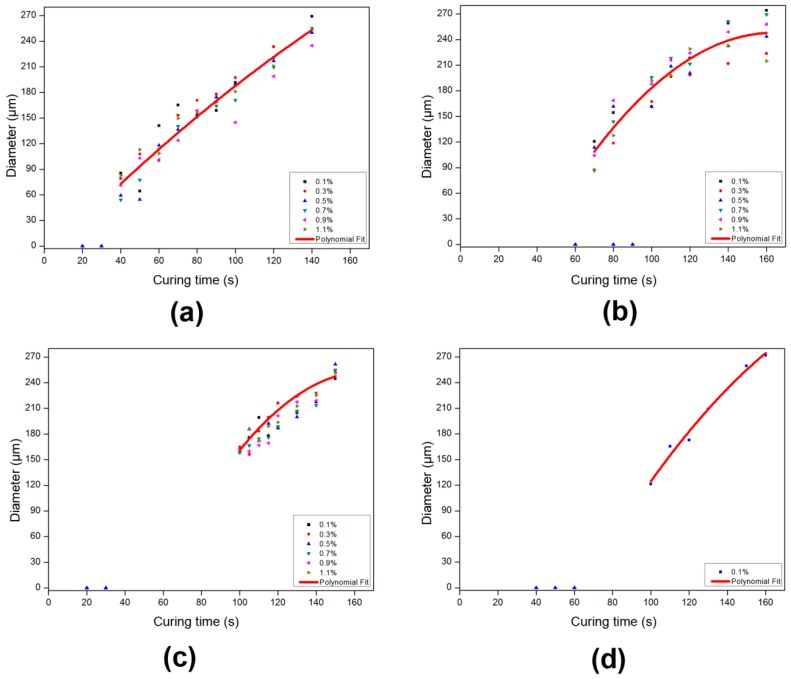
Spacer diameter as a function of curing time for varying inhibitor concentration. Curves correspond to polynomial fits. (**a**) 20% monomer concentration, 2% initiator, inhibitor varied from 0.1% to 1.1%; (**b**) 15% monomer concentration, 2% initiator, inhibitor varied from 0.1% to 1.1%; (**c**) 10% monomer concentration, 2% initiator, inhibitor varied from 0.1% to 1.1%; (**d**) 10% monomer concentration, 1% initiator, 0.1% inhibitor, The diameter of the spacers in the exposure area were measured by optical microscopic – with at least 5 data points averaged to obtain a representative value in each case. Induction times are 40 s, 70 s, 100 s, and 100 s, respectively from Figure 5a–d.

Figure 5 shows the dependence of the spacer diameter on curing time. The initiator and inhibitor content are based on the monomer concentration. The inhibitor was added to improve on the resolution as suggested in the literature for pillar forming photolithographic materials [39,40]. Inhibiting agents stop the polymerization reaction under low intensity conditions and can therefore correct for diffraction effects at the mask edges and eventually present scattering. As we can see from Figure 5a–d, there is an induction time. We define the induction time as the period immediately after the commencement of irradiation until the first observation of spacer formation. No further obvious effect of inhibitor concentration was evident. We anticipate that dissolved oxygen in the water already dominates the inhibition reaction, as evidenced by the induction period at 0.1% inhibitor concentration. As shown in Figure 5 the diameter of the pillar structure depends on the exposure time. When varying the monomer concentration from 20 to 10 wt. % with constant initiator concentration (2 wt. %) all curves overlay (Figure 5a–c) with the exception of the time at which initial structure formation is observed. This induction period in the polymerization reaction is determined by the time that the inhibiting species (oxygen and to a minor extent the inhibitor) is brought below a critical concentration as they react with the generated free radicals of the initiator. From that moment on the propagation reaction of the polymerization starts and the structures are formed. It then also makes sense that the induction period is longer for lower monomer concentration as this is accompanied with lower absolute initiator concentration. When the initiator is further lowered (Figure 5d) the induction period is prolonged further.

The fact that the structures formed at extended curing times can exceed the hole size of the mask is presumably due to diffusion occurring during the polymerization process. The viscosity of the system under which we build up our photolithographic structures is lower than typical photolithographic resists. Therefore, the diffusion of reactive species such as initiator fragments and smaller polymer chains is faster and thus plays a much more prominent role.

### 2.4. Influence of Aperture Size on the Polymerization

**Figure 6:**
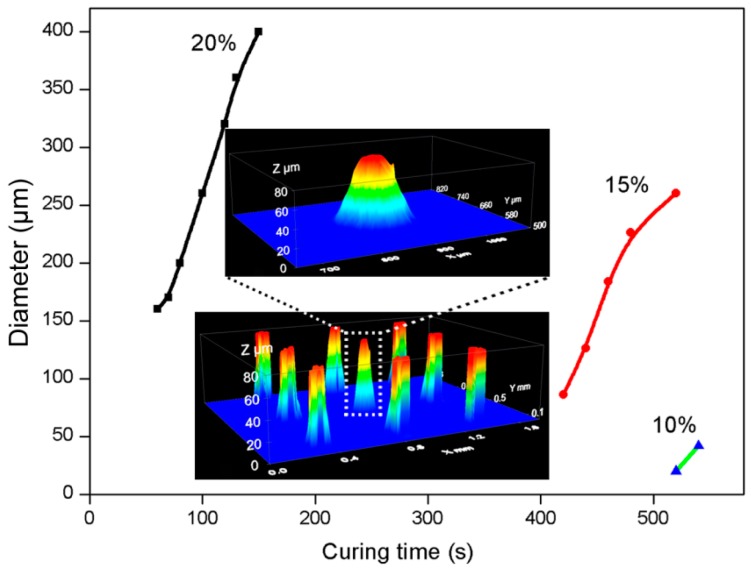
Diameter *versus* curing time for monomer concentrations of 20 wt. %, 15 wt. % and 10 wt. %. 20um spacer mask, pitch 495 μm. Lamp intensity 780 mW/cm^2^, the inset shows 3D surface profiles images taken *ex situ* of spacers on the cover plate (20% monomer concentration, curing time 60 s).

We also conducted a further investigation of spacer formation using a mask design with smaller exposure areas of 20 μm diameter (area fraction ~0.001. The results are shown in Figure 6.

**Figure 7:**
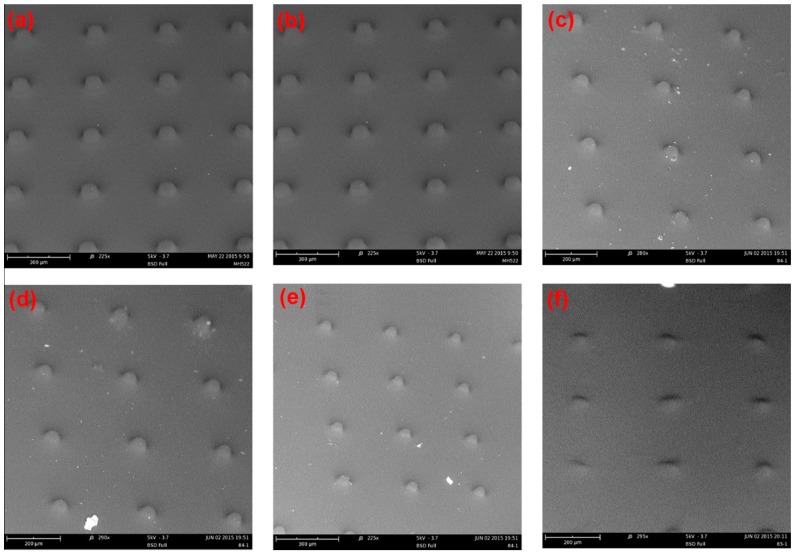
SEM images of spacer arrays formed under various conditions. Water solution: 15% monomer concentration, 2% initiator, inhibitor varied from 0.1% to 1.1%. (**a**) 0.1%; (**b**) 0.3%; (**c**) 0.5%; (**d**) 0.7%; (**e**) 0.9%; (**f**) 1.1%. Lamp intensity 500 mW/cm^2^, Curing Time 400 s; 20 μm mask aperture in this case.

The effect of monomer concentration on spacer diameter was much stronger for small mask aperture. At 20 wt. % spacers much larger than the aperture were observed to form very quickly. Reducing the monomer concentration to 15 wt. % shifted the diameter/curing time considerably, however the initially observed pillars were still larger than the mask aperture. A SEM (Scanning electron microscope) was used to explore the effect of inhibitor concentration at this monomer concentration (Figure 7). One may conclude that the presence of dissolved oxygen, and the rate at which it is consumed by reaction with the forming free radicals, plays here an even more dominant role. At smaller diameter of the forming pillars the excess of oxygen diffusing into the activated area is larger with respect to the number of free radicals formed per unit of time. All of the pillars were observed to have a column shape indicating that the inhibitor content does not affect the shape and only a small amount of inhibitor can maintain the shape in the illuminating area. At the same time, the inhibitor can stop the polymerization in the non-illuminated area. When the inhibitor content was increased to 1.1%, the spacers were not so clearly defined under the SEM. 

It is clear form Figure 6 that we need to reduce the monomer concentration to 10 wt. % to obtain spacers with a diameter corresponding to the aperture size.

## 3. Application in EFD Devices

**Figure 8:**
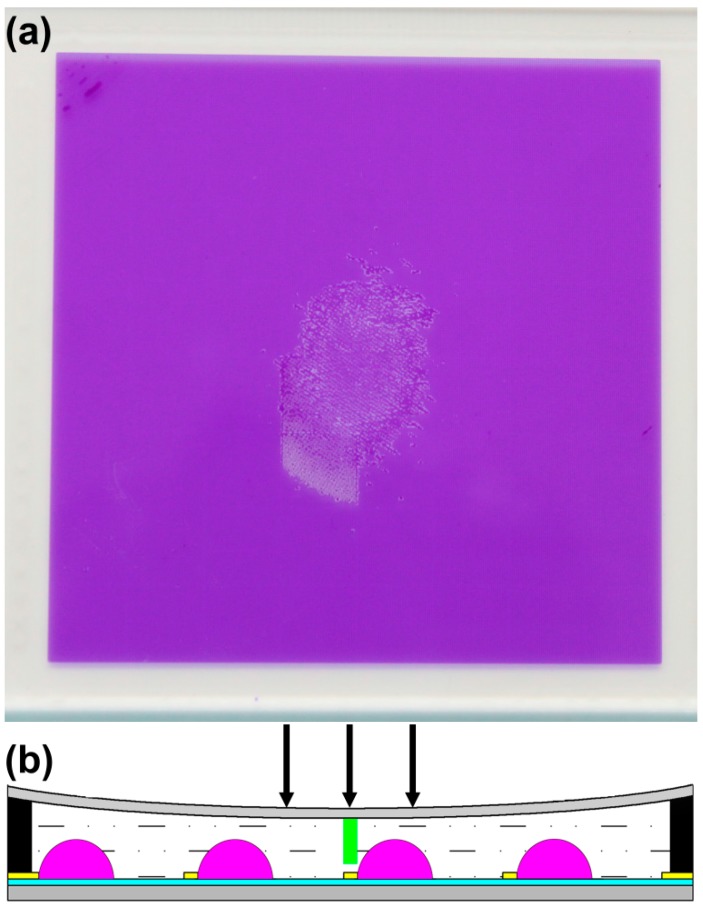
(**a**) mechanical disturbance causes expulsion of oil from pixels in the middle of the plate (90 × 90cm^2^); (**b**) spacer positioning to prevent oil film collapse due to cover plate sag or external pressure.

For EFD devices, the spacers need ideally to be positioned above a fraction of the pixel wall junctions (Figure 8). An accurate alignment machine is needed to position the mask above the cell in the future. In the absence of this alignment facility we made devices filled with colored oil and UV materials (10% monomer concentration, 2% initiator, 1% inhibitor) with 0.2 mm cover plate (adhesive thickness is 75 μm) using manual mask alignment.

**Figure 9:**
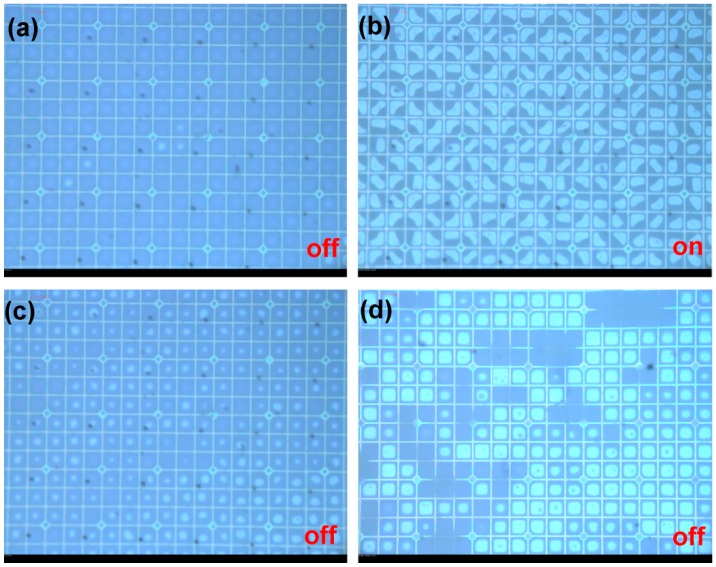
(**a**–**c**) These images show the off/on/off switching cycle with and without voltage (30 V) after the column spacers (indicated by the black dots) are grown in EFD devices; (**d**) the off state showing cell collapse and irreversible oil migration in the area without spacer in the same device.

The thin cover plate was used so as to be very sensitive to mechanical disturbance and correspondingly sensitive to stabilization via our spacer methodology. The transparent areas were an array of 20 μm circles with a pitch of 495 μm on the chromium mask. Curing was performed at a lamp intensity of 780 mW/cm^2^ for 520 s. Spacers of 20 μm diameter were observed to form in the devices (seen as black spots), but of course not over the pixel wall junctions due to manual alignment (Figure 9a). When applying voltage to the device, the oil switched like a standard stable device (Figure 9b,c). 

**Figure 10:**
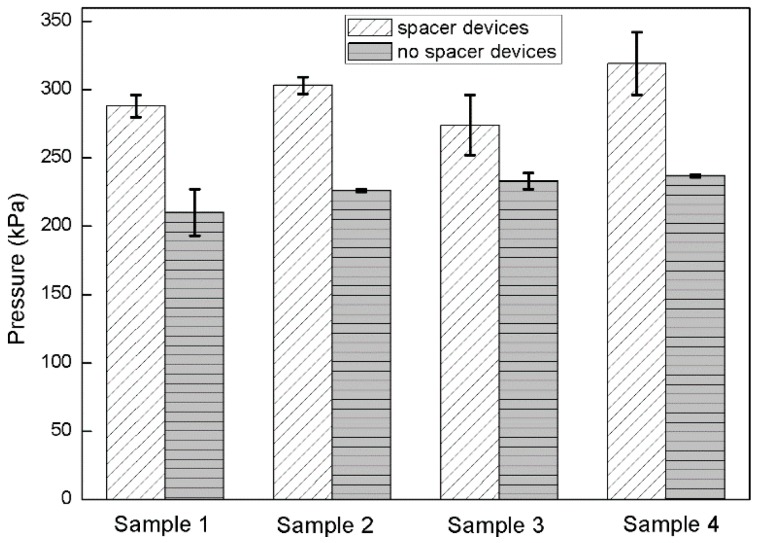
Pressure test of EFD devices with and without spacers. The error bars show the standard deviation from the mean.

A mechanical strength test was done by pressing a metal rod with contact area of 7.8 mm^2^ against the cover plate of the cell supported on an electronic balance until the oil was forced out of the pixel by contact with the cover plate. In this way we were able to measure the force applied to collapse the cell gap. Reference devices without spacers were also tested. The results are shown in Figure 10. The stabilization of the cell gap due to the presence of photo spacers is clearly apparent. Spacers with diameter up to 50 μm were observed to provide additional mechanical stability. This confirms that spacers play a clear role in stabilizing devices against the effects of external pressure.

## 4. Experimental

### 4.1. Materials and Instruments

**Figure 11:**
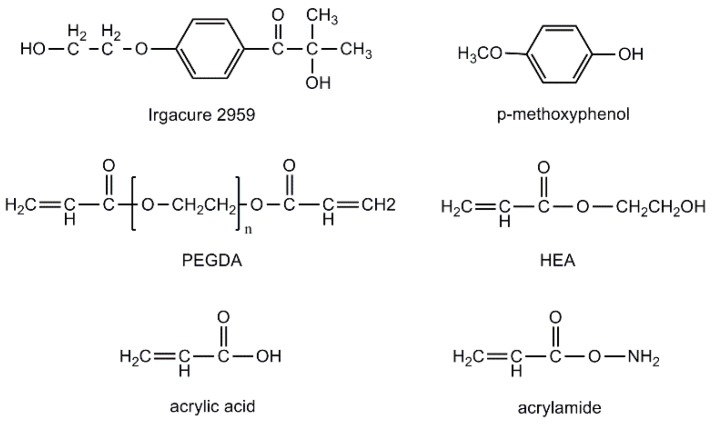
Structures of UV sensitive materials used in this investigation.

Free radical photoinitiator (2-hydroxy-1-[4-(2-hydroxyethoxy) phenyl]-2-methyl-1-propanone) (Irgacure 2959) (purity: 98%, *MW* = 224.25) was used to generate the free radicals for the following chain propagation with monomers after UV illumination. Polyethylene glycol diacrylate (PEGDA) (purity: >92%, *MW* = 250, *ρ* = 1.11 g/mL) was used as cross-linker to propagate with monomers, 2-hydroxyethyl acrylate (HEA) (purity: 96%, *MW* = 116.12, *ρ* = 1.011 g/mL), acrylic acid (purity: 99%, *MW* = 72.06, *ρ* = 1.051 g/mL), acrylamide (purity: 99%, *MW* = 71.08). Photoinhibitor (p-methoxyphenol) (purity: 99%, *MW* = 124.14) was used to inhibit the polymerization in the neighborhood of the illuminating area for providing a suitable column spacer. All of the materials were purchased from Sigma Aldrich except for the acrylamide which was procured from J&K Scientific Ltd (Beijing, China). All of the materials were used directly without further purification (Figure 11). 

UV spot source (OmniCure^®^ S1500, Lumen Dynamics Group Inc., Waltham, MA, America), Mechanical tester (LRPLUS, LLOYD, West Sussex, England), 3D surface profiles (DCM8, Leica, Wetzlar, Germany), SEM (G2 Pro, PHENOM, Shanghai, China).

### 4.2. Preparation of Samples

The monomers, cross-linker and photoinitiator were first dissolved in water. Five solutions of monomer mixture, 20 wt. %, 17.5 wt. %, 15 wt. %, 12.5 wt. % and 10 wt. %, were prepared and the ratio of PEGDA: HEA, PEGDA: acrylic acid, PEGDA: acrylamide were 1:9 in each concentration. 2% photoinitiator (based on monomer concentration) was added. Each formulation was introduced into a 5 mL glass bottle and cured under UV light (110 mW/cm^2^) at 320–500 nm for 600 s to ensure complete polymerization.

### 4.3. In Situ Fabrication Spacer in EFD Cell

**Figure 12:**
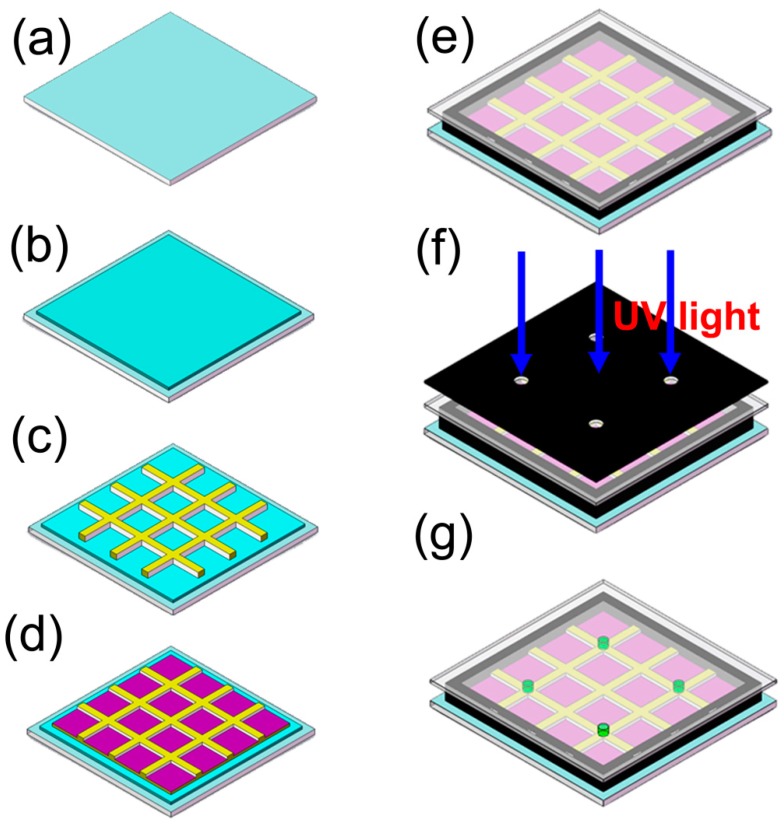
The process for spacer formation in EFD devices, (**a**) cleaning the ITO glass; (**b**) spin coating hydrophobic insulator; (**c**) making the pixel walls with n-type photoresist by lithography; (**d**) adding oil and water solutions onto the engineered substrate; (**e**) sealing the cell with the cover plate/edge seal; (**f**) illuminating the display cell via a patterned mask by UV light; (**g**) forming the column spacer in the selective area.

To construct our EFD devices, we first cleaned the 6’’ top and bottom ITO substrates in a batch cleaning machine involving ultrasonic treatment (40 kHz) with 5% and 3% detergent (RM11-07, Runmon, Shenzhen, China) successively, followed by high purity water spray rinsing, further ultrasonic treatment in water, slow pull from hot ultrapure water, and finally drying at 90 °C for 3 min. Then the fluoropolymer insulator was spin-coated onto the bottom substrate and pixel walls were made using n-type photoresist (e.g., SU-8 photoresist) by lithography. Before introducing the oil and water solutions onto the surface engineered substrate, the mixture of monomer, photoinitiator and photoinhibitor were dissolved in a water solution, stirring for 15 min under yellow light. The monomer concentration is correlated with the density and height of the spacer on the panel. Moreover, the cross-linking degree of the monomer can be controlled by varying monomer concentration, lamp intensity and UV curing time. The cover plate (1.1 mm or 0.2 mm) was chemically cleaned in a UV/O_3_ system (UVOCS) for 15 min before the monomer mixture was sealed into EFD devices with pressure sensitive adhesive (75 μm, acrylate type sourced from Sanwei [HK; Part nr. FP1785-BT]) and irradiated with UV light. The spacers grew in three dimensions in the illuminating regions using a simple mask (Figure 12).

### 4.4. Equilibrium Swelling Behavior

The swelling ratio (SR) was determined by a gravimetric method. Column hydrogel samples (diameter: 20 mm, height: 18 mm) were immersed in ultrapure water and the weight of the fully swollen sample (*W*; g) was measured after carefully removing moisture on the surface with filter paper at the room temperature. The weight of the dried sample (*W*_d_; g) was determined after drying samples at 120 °C until no further weight loss was detectable. The *SR* of samples was calculated as follows [41,42,43]:

(1) $$SR(\%)=(W_{w}-W_{d})/W_{d}\times 100\%$$

### 4.5. Mechanical Properties

The fully swollen samples were used for mechanical testing. After excess water was removed, the samples were tested at the rate of 5 mm/min on a LLOYD mechanical tester, equipped with 100 N load cell. The compressive modulus was determined from the gradient of the stress-strain curves.

### 4.6. Influence of Exposure Time on the Polymerization

A mixture of PEGDA and HEA (1:9) with varying concentration containing 2% initiator and variable inhibitor content was diluted in water. The solution was stirred and sealed into the cell (25 mm × 12 mm) with the adhesive (1.1 mm cover plate). The sample without oil was placed under the mask where it was exposed to the UV spot source. A mask with exposure areas of 0.2 mm diameter, at a separation of 1 mm (area fraction ~0.03) was used. The lamp intensity at the sample position was fixed at 500 mW/cm^2^ and the irradiated area was a 32 mm diameter circle.

## 5. Conclusions

In this article, we introduce a new method to fabricate spacers for switchable electrofluidic optical elements such as display devices, a viable alternative to traditional spacer addition, by using photo induced phase separation. Within minutes the spacer can be formed *in situ* in irradiated areas using UV illumination. It is demonstrated that the cell gap of EFD device can be controlled by PEGDA hydrogels based on polymerizing the co-monomer without affecting either the fluidic or electro-optic properties of the devices. 

## Abbreviations

EFD: electro-fluidic display
LCD: liquid crystal display
ITO: indium tin oxide
PEGDA: polyethylene glycol diacrylate
HEA: 2-hydroxyethyl acrylate
EWOD: electrowetting on dielectric
MEMS: microelectromechanical systems
EWD: electrowetting display
PDLC: polymer dispersed liquid crystal
SEM: scanning electron microscope
SR: swelling ratio
